# Neuroendocrine tumors in a patient with multiple endocrine neoplasia type 1 syndrome: A case report and review of the literature

**DOI:** 10.1097/MD.0000000000034350

**Published:** 2023-07-21

**Authors:** Jian Deng, Xinyi Liao, Hong Cao

**Affiliations:** a Department of Thyroid Breast Surgery, The Second Affiliated Hospital, Hengyang Medical School, University of South China, Hengyang, Hunan, China; b Department of Anesthesiology, West China Hospital, Sichuan University, Chengdu, Sichuan, China.

**Keywords:** case report, MEN-1, NETs, neuroendocrine tumor

## Abstract

Hyperparathyroidism is caused by parathyroid tumors combined with gastroenteropancreatic tumors and pituitary tumors, which is common in patients with multiple endocrine neoplasia 1 syndrome (MEN-1). As its main pathogenic factor involves genetic mutations, it can cause a variety of different clinical symptoms. However, cases with negative genetic testing results and multiple nonfunctional malignant neuroendocrine tumors (NETs) with metastasis are relatively rare.

**Patient concerns:**

A 33-year-old man was admitted to the hospital for hyperparathyroidism. Imaging examination revealed multiple nodules in the parathyroid gland, pancreas, thymus, and adrenal gland, and multiple metastases to the lung, liver, thoracolumbar, as well as mediastinal lymph nodes.

**Diagnoses:**

After multidisciplinary consultation, this patient was diagnosed with MEN-1 syndrome with various original tumors and multiple systemic metastases.

**Interventions:**

The patient underwent parathyroid tumor resection and metastasis biopsy.

**Outcomes:**

The patient received denosumab and sorafenib treatment.

**Lessons:**

As an autosomal dominant hereditary disease, MEN-1 patients present with parathyroid hyperplasia, pancreatic and intestinal tumors, pituitary tumors, and so on, which are caused by genetic mutations. These patients would have hyperparathyroidism, hypoglycemia, gastric ulcer, and gastrointestinal diseases. However, some patients with MEN-1 syndrome cannot be diagnosed by genetic testing and simultaneously present with multiple nonfunctional NETs with systemic metastasis. This increases the difficulty of diagnosis and the subsequent treatment.

## 1. Introduction

MEN-1 is an autosomal inherited syndrome involving multiple endocrine tumors. It manifested by multiple mutations in the tumor suppressor gene MEN-1, which is located on chromosome 11q13. The gene consists of 10 exons and encodes the 610 amino acid menin protein,^[1]^ mutations of which can be used as diagnostic criteria for MEN-1 syndrome. These tumors often occurs in the parathyroid gland, pancreas, anterior pituitary gland and other sites.^[2]^ In recent years, due to the deep understanding of MEN-1, it is also found in the breast, bronchus, uterus, and so on. Most of the reported MEN-1-related neuroendocrine tumors (NETs) were monogenic. Here we report a case of a patient with polygenic MEN-1-related NETs that tested negative for MEN-1 gene, and discuss the clinical presentation and treatment of the syndrome.

## 2. Case presentation

A 43-year-old male was admitted to the Second Affiliated Hospital of Hengyang Medical School on September 6, 2022 due to “the discovery of a left neck mass for 1 month.” The mass was about egg size without pain. Occasional tenderness over back and chest. Ultrasonography suggested a possible bilateral parathyroid adenoma of both side. Parathyroid hormone (PTH) was 701.80 pg/mL (normal range: 18.5–88.0 pg/mL). 2 years ago, he underwent ureteroscopic laser lithotripsy for “left ureteral stone”. His younger brother underwent “surgery for left kidney stone” 1 year ago and “bilateral parathyroid adenoma resection” 3 months ago at our hospital. His father was diagnosed with primary hyperparathyroidism 5 years ago. His uncle underwent partial pancreatic resection at the local hospital 2 years ago and considered pancreatic neuroendocrine adenoma. None of his family members were tested for the MEN-1 gene. The physical examination showed a 4.0 × 4.0 cm mass with indistinct borders and poor mobility was palpable in the left supraclavicular fossa and lateral border of the sternocleidomastoid muscle, no mass was palpable in bilateral thyroid glands and thoracoabdominal examination was normal.

**Figure 1:**
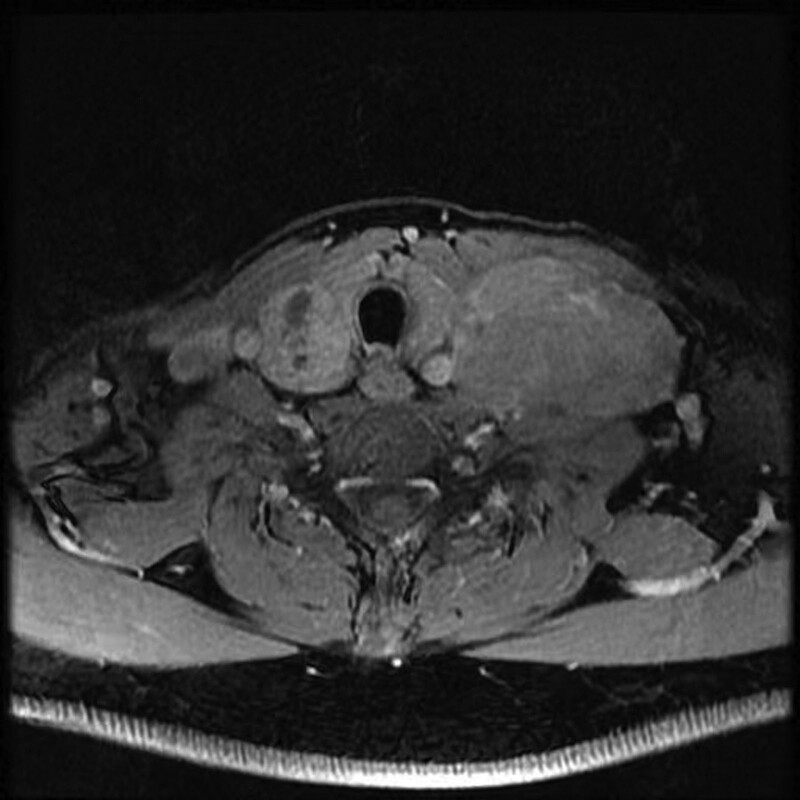
. Magnetic resonance imaging revealed a massive abnormal signal shadow in the bilateral para-thyroid areas. Another mass of abnormal signal shadow in the left neck.

**Figure 2.**
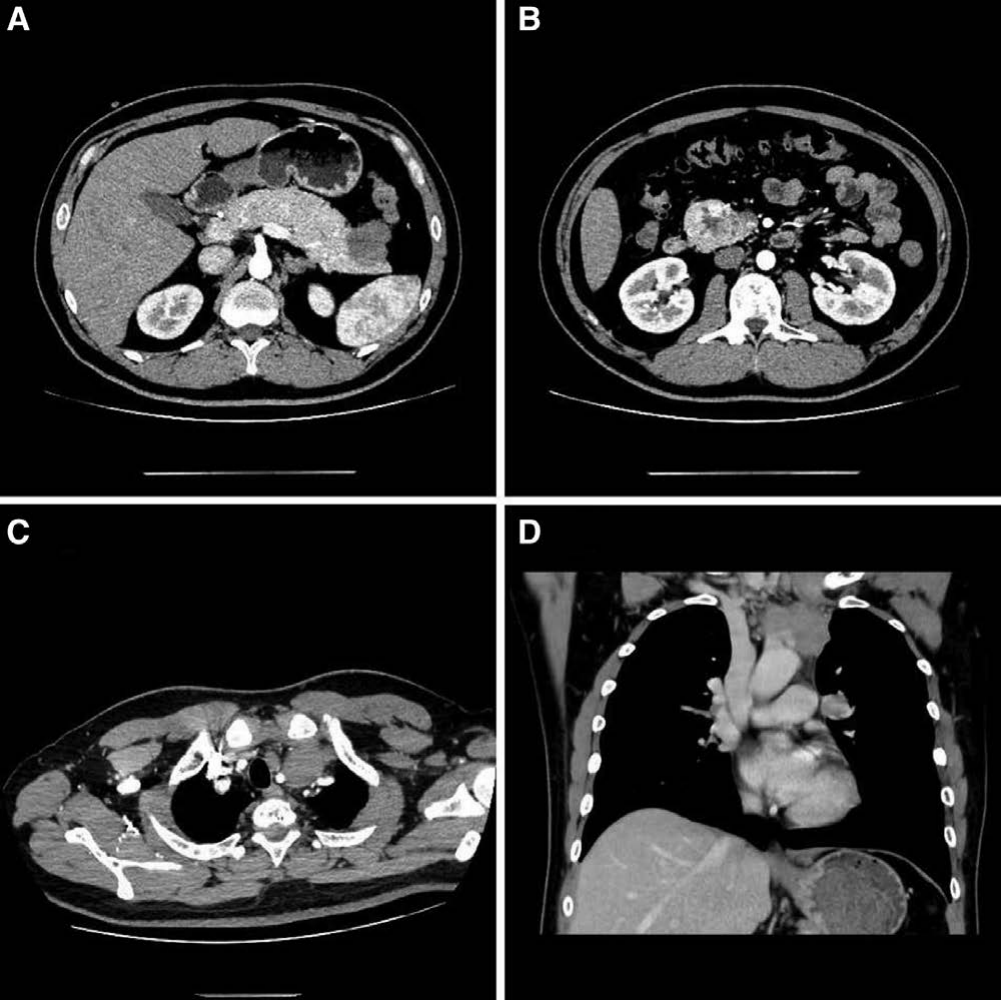
Contrast-enhanced computed tomography scan revealed: (A) Multiple lesions of pancreas. (B) Multiple enlarged lymph node shadows around the pancreas. (C and D) A mass of shadow in the anterosuperior mediastinum.

**Figure 3.**
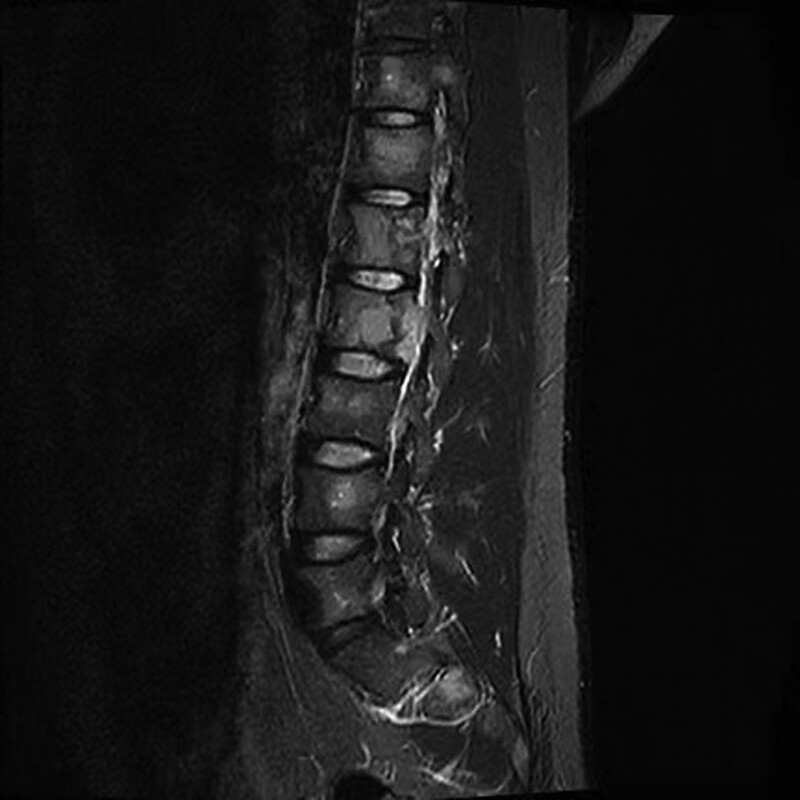
Magnetic resonance imaging revealed abnormal signal shadow of cervical and lumbar vertebrae.

Except the high levels of PTH, serum and urine calcium were reached to 3.13 mmol/L (normal range: 2.25–2.75 mmol/L) and 12.6 mmol/24 h (normal range: 2.5–7.5 mmol/24 h), respectively. Serum concentrations of calcitonin, growth hormone, adrenocorticotropic hormone, gastrin, 25 hydroxyvitamin D, blood glucose were within the normal range, as was thyroid function. Thyroid gland magnetic resonance imaging (MRI) revealed a massive abnormal signal shadow in the bilateral para-thyroid areas (Right, approximately 25 mm × 18 mm × 44 mm; Left, approximately 13 mm × 8 mm × 12 mm), consider the high possibility of a parathyroid adenoma. Another mass of abnormal signal shadow in the left neck, (approximately 61 mm × 37 mm × 63 mm), metastatic tumor awaits exclusion? (Fig. 1). Contrast-enhanced computerized tomography scan (CT) revealed that a mass of soft tissue density shadow in the anterosuperior mediastinum (approximately 40 mm × 30 mm × 37 mm), considered thymoma? metastasis? Multiple pancreatic lesions (head of pancreas, 45 mm × 37 mm × 40 mm; tail of pancreas, 36 mm × 32 mm × 35 mm). Multiple enlarged lymph node shadows can be seen around the pancreas, suggested the possibility of NETs. Multiple nodules in the bilateral adrenal glands (Fig. 2). Extensive bone changes in axial bone, pelvis and upper femur. Thoracolumbar spine MRI revealed abnormal signal shadow of cervical-7, lumbar-5 vertebrae with appendages and sacral 1 to 3 vertebrae. A soft tissue mass in cervical-7 and thoracic-2 vertebral layer was accompanied by spinal cord invasion (Fig. 3). Bone mineral density test: below same age. Cranial MRI was unremarkable.

**Figure 4.**
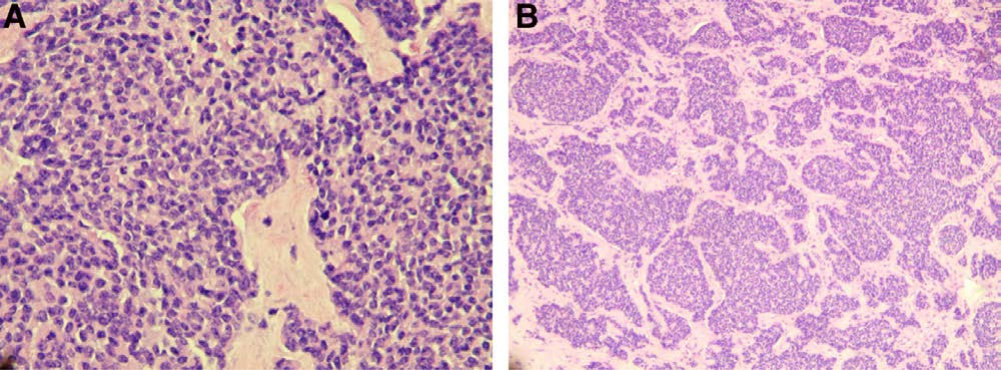
Microscopic (histologic) images of neuroendocrine tumor G3 with small foci of necrosis (A: hematoxylin and eosin, staining, 10× magnification; B: hematoxylin and eosin, staining, 50× magnification).

After the multidisciplinary consultation, combined with the history of the patient, clinical situation, laboratory test, and imaging examination, the possibility of MEN-1 was considered. The patient with severe hyperparathyroidism, hypercalcemia and urinary calculi, while the nature of the cervical and anterior mediastinal mass was unknown. Comprehensive evaluation of the patient condition, bilateral parathyroid adenoma resection + left neck and anterior mediastinal mass resection biopsy was performed. Intraoperative frozen section examination showed bilateral parathyroid adenomas. Left cervical and anterior mediastinal mass suggested the possibility of malignant tumor. Postoperative pathological examination revealed: (Retrosternal mass) NET G3; tumor thrombus was scanned in the vasculature. Immunohistochemical examination: CK7 (−), CAM5-2 (+), CD56 (+), chromogranin A (+), synaptophysin (+), CK5/6 (−), P40 (−), CD5 (−), CD117 (+), E-cadherin (+), CD34 (blood vessel +), Ki-67 (+20%); left parathyroid hyperplasia and right parathyroid adenoma; (left neck) NET G3 with small foci of necrosis. Immunohistochemical examination: CK-pan (+), chromogranin A (+), synaptophysin (+), CD56 (+), insulinoma-associated 1 (+), SSTR 2 (3 +), P53 (wild type), retinoblastoma protein (+), Ki67 (20% <Ki67 <50%), PTH (−), CyclinD1 (partial +) (Fig. 4). Reexamination of PTH was decreased to 2.10 pg/mL and blood calcium was normal. The patient tested negative for the MEN-1 gene. Postoperative period was treated with oral calcium, vitamin D to prevent hypocalcemia. The patient recovered well with no hoarseness and low calcium manifestations such as numbness of hands and feet.

Continued refinement of 18F-FDG-PET/CT revealed: Pancreatic head and tail mass with high FDG metabolism and rich blood supply to the pancreatic head for consideration of primary NET; multiple bone metastases in both lungs, pleura and whole body; the possibility of metastasis to the right supraclavicular, mediastinal and both hilar lymph nodes; Bilateral adrenal nodular coarsening, small nodules in the hepatic capsule, no increased FDG metabolism was observed. 68Ga-SSA- PET/CT: Left anterior superior mediastinal mass with mildly increased NOC uptake and was considered for NET; multiple lymph node metastases in mediastinum, bilateral hilum, left internal mammary gland, section mildly expressing SSTRs; bilateral pulmonary and pleural metastases, extensive and mixed systemic bone metastasis, increased SSTR expression to varying degrees, and high FDG metabolism; Multiple slightly hypodense shadow in pancreas, increased uptake of NOC in different degrees, low metabolism of FDG, was considered as multiple NETs (primary of pancreas?), partly showed high expression of SSTRs; Left adrenal nodule, no increased FDG metabolism and expression of SSTRs, was considered the possibility of adenoma.

After the secondary multidisciplinary consultation, experts considered this patient as MEN-1 syndrome, combined with thymus, pancreas and other multiple NETs of various origins with multiple systemic metastases, parathyroid adenoma, adrenal tumor. Denosumab was given for bone metastases, and sorafenib was given for NET (300 mg each time, once a day. Treatment cycles were continued until disease progression or intolerable toxicity).

## 3. Discussion and conclusions

Multiple endocrine neoplasia (MEN) is a rare syndrome in which tumors occur simultaneously or sequentially in multiple endocrine organs. It can be classified into MEN-1, MEN-2, MEN-3, and MEN-4 based on pathogenesis and clinical manifestations.^[3]^ MEN-1 also known as “Wermer syndrome.” It was first recognized in the early 20th century and was mostly characterized by associated tumors of the parathyroid, pancreas, pituitary, etc. The syndrome is inherited in an autosomal dominant manner, with the morbidity of 1/30,000.^[2]^ First degree relatives of MEN-1 patients have a 50% risk of developing the syndrome.^[3]^

It is believed that the emergence of the MEN-1 syndrome is mainly due to a disruption of menin protein translation caused by mutations in the tumor suppressor gene MEN-1. As a scaffold protein, menin could increase or decrease gene expression through epigenetic regulation of gene expression by histone methylation, which in turn participated in transcriptional regulation, genome stability, cell division and proliferation.^[4]^ Regarding the pathogenesis of MEN-1 syndrome, it is currently accepted as the “twice hit theory” proposed by Kundson, that is, the patient inherits 1 mutated allele of MEN-1 from a parent but fails to develop the disease because the other allele is normal. Once the other MEN-1 allele is also mutated at the somatic level, then the disease would occur.^[5]^ Therefore, genetic screening for MEN-1 is helpful to confirm the clinical diagnosis and to monitor the tumor in early stage. However, not all MEN-1 patients have mutations in this gene, and approximately 10% to 30% of MEN-1 patients test negative for it and can only be diagnosed based on clinical manifestations, imaging examination and other information. These patients may have mutations within the promoter region or the untranslated region of the MEN 1 gene.^[6]^ It could also be that there is another undiscovered tumor suppressor gene in this region.^[7]^ A Dutch study of 293 patients with MEN-1 syndrome showed that patients with negative MEN-1 mutations had less aggressive tumors than patients with positive MEN-1 mutations. The median survival rate in the MEN-1 gene mutation-negative patient group was 14 years higher than that in the MEN-1 mutation-positive patient group.^[8]^

According to statistics, the mean age at onset for MEN-1 patients is 20 to 25 years old, and 95% of them develop clinical symptoms before the age of 50. Its clinical characteristics can be manifested as different combinations of more than 20 endocrine and non-endocrine tumors, which can cause damage to the body by the excessive secretion of hormones or by the sustained growth of the tumor itself.^[9]^ Data have shown that more than 95% of MEN-1 patients have primary hyperparathyroidism, followed by enteropancreatic NETs (50%) and pituitary tumors (40%). Diagnosis of MEN-1 was also based on the occurrence of tumors in 2 of the above 3 major sites, or in 1 of the 3 major sites in first-degree relatives of MEN-1 patients, or the presence of germ-line MEN-1 mutations in asymptomatic individuals.^[10]^ This patient presented with hyperparathyroidism and multiple NETs of pancreatic and thymic origin, which is extremely rare.

Most MEN-1 syndrome patients present with primary hyperparathyroidism as the first clinical manifestation, often showing entire parathyroid hyperplasia. Its related symptoms mainly include hypercalcemia, kidney stones, osteoporosis and so on. Another syndrome with similar manifestations is familial isolated hyperparathyroidism, which is characterized by isolated hyperparathyroidism without pituitary or pancreatic tumors, and has also been suggested to an early manifestation of MEN-1.^[6]^

Surgery is the preferred treatment for MEN-1-related hyperparathyroidism, but the scope of surgery remains controversial. Because the lesion often involves the entire parathyroid gland, it is usually necessary to resect all glands and selectively perform partial parathyroid autotransplantation.^[3]^ However, it may take years or longer to achieve compensation in some patients, and permanent hypoparathyroidism may occur due to autologous transplantation failure.^[11]^ This is associated not only with increased risk of renal insufficiency, malignancies, cardiovascular accidents, but also with increased mortality.^[12]^ Some scholars suggest that part of parathyroid tissue can be cryopreservation, and autologous parathyroid transplantation can be performed again within 12 months.^[13]^ An alternative surgical approach is subtotal parathyroidectomy (general excision of 3–3.5 parathyroid glands), which is less likely to produce permanent hypoparathyroidism, but may occur recurrent primary hyperparathyroidism in about 13% of patients. The absence of precise preoperative positioning is the main cause of the failure to accurately resect the parathyroid tissue. Kartini et al^[12]^ suggested that the choice of subtotal resection of parathyroid adenoma should be considered only if there is precise localization of parathyroid glands on ≥2 imaging modalities with consistent results. Furthermore, parathyroid glands within the thymus are present in 15% to 20% of patients with MEN-1. If 4 parathyroid glands cannot be found intraoperatively, performing thymotomy along with parathyroidectomy is also an option to identify additional parathyroid glands. It is beneficial for the early identification of thymic carcinoid, but it increases the risk of adjacent phrenic nerve and recurrent laryngeal nerve injury.^[14]^ Interventional procedures such as absolute ethanol injection and electrical ablation are also applied in the clinic, but more clinical evidence is needed to prove their safety and efficacy.

Pancreatic neuroendocrine tumor (PNET) is the second common tumor of MEN-1 syndrome and the most common cause of death in patients with MEN-1.^[15]^ About 50% of MEN-1 patients would develop PNET by the age 50, and nearly 90% by the age 80.^[16]^ Therefore, early examination and early intervention of PNET appear to be particularly important. PNET can be classified into functional and nonfunctional PNET according to whether excess hormone is produced or not. Nonfunctional PNET accounts for the majority of MEN-1 patients, at approximately 60% to 90%.^[17]^ The World Health Organization classified gastroenteropancreatic NETs into highly differentiated NETs and poorly differentiated neuroendocrine carcinoma based on proliferation markers (Ki-67 and mitotic counts). The highly differentiated NETs were further divided into G1, G2, and G3. Poorly differentiated neuroendocrine carcinoma is very similar to highly differentiated NET G3, showing poor differentiation and poor prognosis. Although these tumors are growing slowly, but they may rapidly develop distant metastasis. The data showed that the median survival for patients with localized nonfunctional PNET was 240 months. Overall survival decreased to 90 months in patients with localized tumors and 25 months with unresectable metastatic tumors.^[18]^

Surgery is both a treatment and a prevention. However, there are different insights on the time and extent of surgical intervention, mainly depending on the size and location of the tumor. Existing studies have shown that larger tumors have higher odds of metastasis and lower rates of patient survival.^[19]^ According to European Neuroendocrine Tumor Society recommendations, tumor with diameter exceed 2 cm, infiltration or positive lymph node should undergo lesion resection and regional lymph node dissection. Smaller tumors can be closely monitored, and 80% of small PNET can remain relatively stable for 10 years.^[20]^ However, when conditions permit, the nature and differentiation of the tumor should be evaluated by puncture biopsy. The location of the tumor will determine whether patients need total pancreatectomy, pancreatic tail or body resection or Thompson surgery, etc. Thompson surgery includes pancreatic body and tail resection as well as enucleation of pancreatic head tumor to avoid total pancreatectomy.^[21]^ In a non-randomized and non-blinded study, somatostatin analog lanretide improved progression-free survival in patients with non-metastatic PNET <2 cm.^[22]^ However, stronger evidence is needed before it can be applied to clinical practice.

Liver is the major site of metastasis for nonfunctional PNETs. When patients developed liver metastases, surgical resection, microwave ablation or radiofrequency ablation and peptide receptor radionuclide treatment can be selected as treatment methods.^[23]^ In metastatic patients, systemic drug therapy can be divided into 2 main categories: biologic therapy and chemotherapy. Most biologics are targeting SSTRs,^[24]^ including octreotide, lanretide, etc. Other biological inhibitors such as everolimus, sunitinib have also been shown to be useful in this syndrome. Chemotherapeutic drugs mainly include streptozotocin, temozolomide, and capecitabine.^[25]^ However, due to the rarity of MEN-1 syndrome, detailed clinical trials are currently lacking to be supported.

Combined with clinical manifestations, pathology and imaging examination, the thymic neuroendocrine tumor (TNET) of this patient was considered as a secondary primary tumor. Unlike gastroenteropancreatic NETs, MEN-1-associated TNET rarely present with a functional hormonal syndrome, which are extremely aggressive and prone to invasion and metastasis.^[26]^ According to statistics, the 5-year survival rate of TNET patients was 62.5%, and the 10-year survival rate was only 31.3%.^[27]^ The size of the primary tumor is one of the major prognostic factors in patients with TNET, and the larger the tumor, the worse the prognosis.^[27]^ MEN-1 related TNET is strongly heritable.^[28]^ This also suggests the importance of early screening to identify TNET in family members with MEN-1 syndrome. If TNET is diagnosed by early CT screening, the course of the disease and the patient survival rate are significantly better than if significant clinical symptoms are already present. Moreover, radical surgery and complete resection for TNET were shown to be associated with better survival rate.^[29]^ Therefore, some scholars have suggested that prophylactic removal of thymus during parathyroidectomy may reduce the risk of death in MEN-1 patients.^[1]^ But even in the case of complete resection, the prognosis would poor because of the aggressive behavior of TENT and the high incidence of tumor recurrence after surgery. At present, peptide receptor radionuclide treatment has good effective for metastatic NETs. Using small peptides combined with radionuclides emitting β radiation, tumor cells absorb drugs and radionuclides that can be killed by emitted β particles.

After a multidisciplinary consultation of this patient, the expert recommended a novel multi-target tyrosine kinase inhibitor (vantinib) to control tumor progression. A multicenter clinical trial of 81 patients (42 PNET, 39 non-PNET) treated with solantinib had higher objective response rate (19%) and median progression-free survival (21.1 months) in the PNET group than in the non-PNET group.^[30]^ Sofantinib has shown good antitumor activity and safety in patients with metastatic NETs.

In recent years, although great progress has been made in studying the diagnosis and treatment, as well as related tumor pathophysiology of MEN-1. However, the manifestations of MEN-1 are diverse and can be easily misdiagnosed. 50% to 70% of patients with MEN-1 syndrome still died from tumor progression.^[31,32]^ Therefore, it is particularly important to improve the awareness of early diagnosis of MEN-1 syndrome, actively prevent tumor progression and carry out multidisciplinary management mode, so as to improve the survival rate of patients with MEN-1 syndrome.

## 4. Limitations

This study had several limitations. First, pathological examination of the primary lesion of the pancreatic tumor was not performed because the patient declined to undergo surgical biopsy. Second, the results included were limited to full-text articles published in English; thus, other reports may exist.

## Author contributions

**Conceptualization:** Hong Cao.

**Formal analysis:** Xinyi Liao.

**Writing – original draft:** Deng Jian.

**Writing – review & editing:** Hong Cao.
